# Spatial Associations Between Contaminated Land and Socio Demographics in Ghana

**DOI:** 10.3390/ijerph121013587

**Published:** 2015-10-27

**Authors:** Russell Dowling, Bret Ericson, Jack Caravanos, Patrick Grigsby, Yaw Amoyaw-Osei

**Affiliations:** 1 Pure Earth, Formerly Blacksmith Institute, 475 Riverside Drive, Suite 860, New York, NY 10115, USA; E-Mails: Bret@pureearth.org (B.E.); Patrickgrigsby@gmail.com (P.G.); 2 School of Public Health, City University of New York, 2180 Third Ave, New York, NY 10035, USA; E-Mail: jcaravan@hunter.cuny.edu; 3 Green Advocacy Ghana, P.O. Box SK 482, Sakumono Estates, Tema, Ghana; E-Mail: wayoma59@hotmail.com

**Keywords:** Ghana, contaminated sites, socio demographics, environmental justice

## Abstract

Associations between contaminated land and socio demographics are well documented in high-income countries. In low- and middle-income countries, however, little is known about the extent of contaminated land and possible demographic correlations. This is an important yet sparsely researched topic with potentially significant public health implications as exposure to pollution remains a leading source of morbidity and mortality in low-income countries. In this study, we review the associations between several socio demographic factors (population, population density, unemployment, education, and literacy) and contaminated sites in Ghana. Within this context, both correlation and association intend to show the relationship between two variables, namely contaminated sites and socio demographics. Aggregated district level 2010 census data from Ghana Statistical Service and contaminated site location data from Pure Earth’s Toxic Sites Identification Program (TSIP) were spatially evaluated using the number of sites per kilometer squared within districts as the unit of measurement. We found a low to medium positive correlation (*ρ* range: 0.285 to 0.478) between contaminated sites and the following socio demographics: higher population density, higher unemployment, greater education, and higher literacy rate. These results support previous studies and suggest that several socio demographic factors may be reasonably accurate predictors of contaminated site locations. More research and targeted data collection is needed to better understand these associations with the ultimate goal of developing a predictive model.

## 1. Introduction

In high-income countries there is a well-documented spatial association between contaminated land and socio demographics. In the United States (US) several studies that have evaluated census tracts proximately located to hazardous waste sites have found significant correlations to poverty and race. In one such study, Kearney and Kiros (2009) found a strong association between high poverty and minority census tracts and locations on the US Superfund National Priorities List (NPL), a grouping of those locations targeted for environmental remediation [1]. Similar studies have been carried out elsewhere—see Burnwell-Naney *et al.* (2009) and Ringquist (2005) for example [2,3]. Burnwell-Naney *et al.* [2] concluded that the majority of black residents (55.9%) and the majority of residents living below the poverty line (57.2%) in the state of South Carolina were located in Superfund host census tracts with hazardous waste sites on the National Priorities List. Additionally, Ringquist conducted a meta-analysis of 49 environmental equity studies and concluded that there is ubiquitous evidence of environmental inequities based on race.

In contrast to these well-document associations in high-income countries such as the US, very little is known about the prevalence and distribution of contaminated land in low-income and medium-income countries, though similar associations are expected. By comparison, the related issues of contaminated air and water have been much more fully documented. Biological contamination of water supplies in low- and middle-income countries led to nearly 850,000 deaths from diarrheal disease in 2014 [4]. Similarly, recent research by WHO indicates that ambient and household air pollution were responsible for seven million deaths in 2012, with the vast majority of these deaths occurring in low- and middle-income countries [5].

The United States Environmental Protection Agency (USEPA) defines environmental justice as “the fair treatment and meaningful involvement of all people regardless of race, origin, or income with respect to the development, implementation, and enforcement of environmental laws, regulations, and policies” [6]. The environmental justice movement famously began in the 1980s in Warren County, North Carolina (USA) with community opposition to the construction of a hazardous waste landfill in a low-income minority area. Since then, it has significantly increased awareness of and attention to the potential correlations between socio demographics and environmental health [7]. Indeed, strong positive associations have been identified between low-income households and proximity to industrial areas in the US (see Maantay, 2001, for example) [8]. Marginalized populations residing on or adjacent to contaminated land can be exposed to a large range of toxicants that negatively impact their physical or mental health. This can lead to a cyclical process of environmental injustice and marginalization whereby low-income poorly educated populations become sicker and, therefore, less able to lift themselves out of poverty [9].

While efforts to determine the extent of contaminated land in low- and middle-income countries are still very much nascent, notable progress has been made in the past decade. Some countries in this income grouping, most significantly Mexico, have developed impressive databases of contaminated sites. Others, like Peru, for example, have begun to make considerable investments in this area. Multinational efforts have largely been organized around a specific contaminant or set of contaminants. The many countries that are signatories to the Stockholm Convention for example, which has been in effect since 2004 and aims to protect human health and the environment from persistent organic pollutants (POPs), have made varying degrees of progress in at least cataloguing locations and quantities of POPs, if not assessing the potential human health risk or exposures. Multiple NGOs and research institutions have collaborated to investigate arsenic contaminated drinking water in South and Southeast Asia, though even here, estimates of exposed populations vary by an order of magnitude [10]. Finally, much work has been done or is beginning to be done on artisanal small-scale gold mining (ASGM) locations, spurred in part by the Minimata Convention. Other efforts exist, but these are among the more notable.

Likely the most comprehensive database to date in this area is that maintained as part of the Toxic Sites Identification Program (TSIP) of Pure Earth, formerly Blacksmith Institute [11]. As of June 2015, 3206 sites in 49 countries have been identified, putting an estimated population of 63 million people at risk. Trained research and site investigators have visited 2,301 sites thus far. Heavy metals represent the greatest portion of pollutants found in the TSIP database. Persistent organic pollutants (POPs) and pesticides also represent a sizeable portion of the database. Using data collected as part of this effort Chatham-Stephens *et al.* (2013) estimate as many as 828,000 Disability Adjusted Life Years (DALYs), a measure of overall disease burden that incorporates both years of life lost and years of life disabled, result from exposures to contaminated sites in India, Indonesia and the Philippines alone [12]. The estimates indicate that terrestrial pollution poses a significant and credible health risk in low- and medium-income countries.

Risk assessment of chemical hazards focuses on addressing pathways of human exposure, namely inhalation, ingestion, or dermal contact. To confirm this pathway, environmental or biological sampling must be conducted, and detailed information about the pollutant, relevant pathway, and population impacted must be gathered. USEPA uses a similar, albeit more robust, model to assess contaminated sites in the United States. This USEPA Hazard Ranking System (HRS) also requires information on spatial attenuation, pollutant persistence, migration potential, and likelihood of future release. Gathering all of this information can be prohibitively costly and time consuming in low- and middle-income countries. Within this context, the TSIP was developed to carry out rapid risk assessments that are cost-effective in low-resource settings [13].

Sites are identified through several methods including knowledge and expertise of local staff, investigation of previously identified legacy sites, collaboration with local governments or research organizations, and nominations. In Ghana, Pure Earth has a strong partnership with a local NGO and a good relationship with both the Ghana Environmental Protection Agency (EPA) and Ghana Health Service. Therefore, the majority of sites in Ghana found in the TSIP database are a direct result of local knowledge, partnerships, and expertise. Once a potential site has been identified, a network of local environmental investigators is utilized to conduct site investigations, and the data is reviewed by an experienced environmental health professional for quality control before approval. All assessed sites go through several levels of formal review with ongoing communication between field staff, research staff, and quality control staff to identify and rectify any inconsistencies or inaccuracies in the data. The program focuses entirely on sites where there exists a reasonable suspicion that point source pollution might be affecting human health. Initial lists of sites that meet this general description are developed, and visits are scheduled to sites based on their presumed severity.

**Figure 1:**
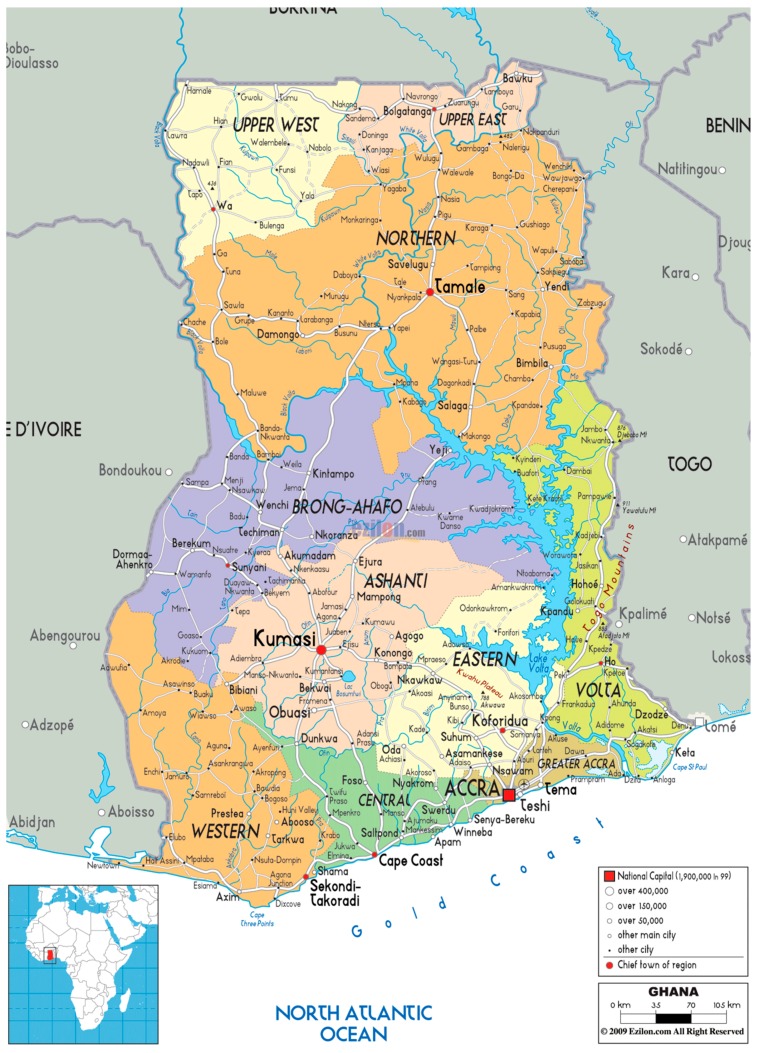
Regional map of Ghana. Source: MAGELLAN Geographics, 1992 [15].

Ghana is a lower middle-income West African country with a well-developed heterogeneous industrial base. The country is home to 25 million residents, the vast majority of whom live in a number of densely populated cities along the southern coast (see Figure 1). Chemical production and metals smelting and processing are among the largest contributors to the formal industrial economy, while informal car battery recycling, artisanal small-scale gold mining, and scrap recycling are industries of most interest in the informal sector.

The Ghanaian government passed Statistical Service Law 135 in 1985, which established the Ghana Statistical Service (GSS) as part of the Ghana Public Service. GSS conducts censuses every ten years and produces a number of reports on topics such as poverty and income at the regional level. Census data is presented in aggregate level by district at Ghana Statistical Service website where it is freely available. GSS is a well functioning public service organization in the Ghanaian government that records and analyzes high quality demographic data [14].

There are currently 217 contaminated sites located in Ghana according to the TSIP database. See Table 1 for a complete breakdown of number of sites by key pollutant in Ghana. A Regional Program Director based in New York oversees a country coordinator in Ghana, who, in turn, manages site investigators to collect the data that is entered into the database. This information is reviewed by at least two quality control personnel who confirm sufficient completion of crucial fields such as key pollutant and sampling test results before approving the site.

**Table 1:** Breakdown of contaminated sites in Ghana found in the TSIP database by key pollutant.

Key Pollutant	Number of Sites
Mercury	77
Lead	53
Other *	55

* A number of “other” contaminants can be found in Pure Earth’s TSIP database including but not limited to arsenic, cadmium, chromium, pesticides, and polychlorinated biphenyls (PCBs). Key pollutant data from 32 sites was not used in this analysis. These sites were either flagged as being either outside the scope of Pure Earth’s work or having results below the recommended level.

Mercury and lead are the two most common pollutants found in Ghana. Mercury exposure in Ghana is often a result of artisanal small-scale gold mining (ASGM), which is the largest contributor to global anthropogenic mercury in the atmosphere [16]. Workers and their families can be exposed to dangerously high levels of mercury vapor throughout the gold mining process. ASGM uses elemental mercury, which poses significant risks for human health since mercury is a potent neurotoxicant [17]. Lead exposure in Ghana can occur from a number of sources including lead smelting, used lead-acid battery recycling, lead mining, and electronic waste recycling. Lead particles in soil and dust can be harmful to humans when ingested or inhaled, particularly to children less than six years. Lead poisoning can cause damage to the brain and nervous system, developmental delays, liver and kidney damage, and, in extreme cases, death [18].

Heavy industry, scrap and electronic waste recycling, and mixed industrial estates are also responsible for many of Ghana’s contaminated sites. The country has a large informal sector where young and typically poor workers are exposed to an array of chemicals without adequate personal protective equipment or occupational standards. Furthermore, children are often exposed to pollutants via soil inhalation or ingestion when informal work is carried out at or adjacent to residential areas. This has serious public health implications because children are the most vulnerable to chemical exposure due to their unique activities, physiology, and critical windows of development [19].

While the TSIP database is likely the most comprehensive database of global contaminated sites to date, it is also necessarily an underestimation. Locating, sampling, tracking, and confirming contaminated sites in low- and middle-income countries remains both a time-consuming and costly endeavor. Furthermore, informal sites are difficult to identify and access, and sites must be re-evaluated at regular intervals to ensure quality of the data. Therefore, TSIP is not a comprehensive database of all contaminated sites globally, but rather is an effort to begin to understand the scope of the problem.

A number of factors potentially support the assumption that poverty and socio economic marginalization would be spatially associated with contaminated sites in low- and medium-income countries. Foremost among them is widespread informality in housing and industry. In the US, widespread formality and well-developed real estate markets largely separate residential and industrial zones. However, in many low- and middle-income countries including Ghana, informal industry results in polluting activities occurring directly adjacent to residential areas. This is particularly apparent in the small-scale informal sector, where families often work directly in their home or backyard. Because there is a well-documented association between informality and poverty, we anticipated a strong positive correlation between pollution and poverty.

## 2. Background and Methods 

Ghana is divided into ten regions that, since 2012, contain 216 administrative districts. The number of districts found in each region can be seen in Table 2.

**Table 2:** Regional divisions of administrative districts and number of contaminated sites identified.

Region	Population per Region	Number of Districts	Number of Sites Within Each Region
Ashanti	4,780,380	30	35
Brong-Ahofo	2,310,938	27	9
Central	2,201,863	20	8
Eastern	2,633,154	26	27
Greater Accra	4,010,054	16	29
Northern	2,479,461	26	4
Upper East	1,046,545	13	5
Upper West	702,110	11	0
Volta	2,118,254	25	1
Western	2,736,021	22	29

In an effort to better understand possible associations between socio demographics and contaminated sites, we utilized Geographic Information Systems (GIS) software (ArcGIS 10.2–Redmond, Esri Inc, 2013, CA, USA) to overlay contaminated site data from the TSIP database with district level data from the Ghana Statistical Service (GSS). SPSS software (SPSS V22.0–Armonk, IBM, 2013, NY, USA) was utilized to analyze the correlation between contaminated sties and the socio demographic indicators. Out of the 217 sites in Ghana, 69 sites were not included in this study because they did not have adequate global positioning system (GPS) data. Therefore, a total of 147 sites were overlaid on the demographic maps.

The unit-hazard coincidence method begins by selecting a geographical unit, such as a zip code, census block, or, in this case, the Ghanaian districts as defined by GSS as the analytical unit. The number of waste sites within this unit is then correlated to the particular demographic attribute of interest. The primary criticism associated with the unit-hazard model centers around the difficulty in accessing proximity to a waste site and therefore exposure impact. Given this study was not correlating any human disease outcomes, we felt the application of unit-hazard was appropriate and offered the best comparison to reported demographic statistics. Essentially, the specific location of a contaminated site within the district was not a necessary parameter. The total number of sites per district was our primary metric. However, to ensure an appropriate application, we also calculated a contaminated site variable per unit area (number of sites per square kilometer for that district).

For this review, five indicators were examined: population, population density, unemployment, education, and literacy rate. These factors were chosen due to the limited availability of administrative district level statistics collected by Ghana Statistical Service. GSS does not collect information on poverty or race, for example. Specific attributes of each socio demographic factor can be found in Table 3. If the data predated the current administrative boundaries, they were fit into the current categories by manually reviewing pre-2012 boundaries and comparing them to today’s boundaries. Administrative district names were edited according to the current system and checked for quality control.

**Table 3:** Census variables used to evaluate demographic characteristics and their associations with contaminated sites.

Socio Demographic Factors	Attribute
Population	Number of persons within a district in 2010
Population Density	Number of persons per kilometer squared within a district in 2010
Unemployment	Percent of Unemployed Adults Age 15–64 within a district in 2010
Education	Percent of persons with a tertiary (bachelor) degree or higher within a district in 2010
Literacy Rate	Percent of persons 11 years and older who are literate within a district in 2010

To review the correlation between the location of contaminated sites and the five socio-demographic categories, the number of sites per square kilometer in each district was calculated and checked to ensure quality control. A Spearman’s rank correlation coefficient (*ρ*) was utilized to measure the degree of positive association between the variables (demographics and contaminated sites). The Spearman correlation was chosen over the standard Pearson correlation due to the non-linearity of the variables. The correlation was calculated in IBM SPSS version 22.0. This exercise was repeated a second time in Excel version 12.3.6 to ensure quality control.

## 3. Results and Discussion

The results of the Spearman correlation can be found in Table 4. It can be assumed that a correlation of less than 0.30 denotes a negligible or at best low correlation between any two variables [20]. Therefore, there is a low positive correlation between higher population and contaminated sites at the administrative district level in Ghana (*ρ* = 0.285). However, for the remaining four socio-demographic factors analyzed in this paper, a low to medium positive correlation is seen. Greater population density, literacy and unemployment are all correlated to contaminated sites at levels above 0.45 (*ρ* = 0.456, 0.463, and 0.478, respectively). Furthermore, education correlates to contaminated sites at a level above 0.40 (*ρ* = 0.412).

**Table 4:** Correlation results.

Socio-Demographic Factors	Spearman Correlation with Number of Sites in Each District Per Km^2^ (Non-Parametric) *
Population	0.285
Population Density	0.456
Unemployment	0.478
Education	0.412
Literacy Rate	0.463

* Cohen’s standard effect size for correlation coefficient.

**Figure 2:**
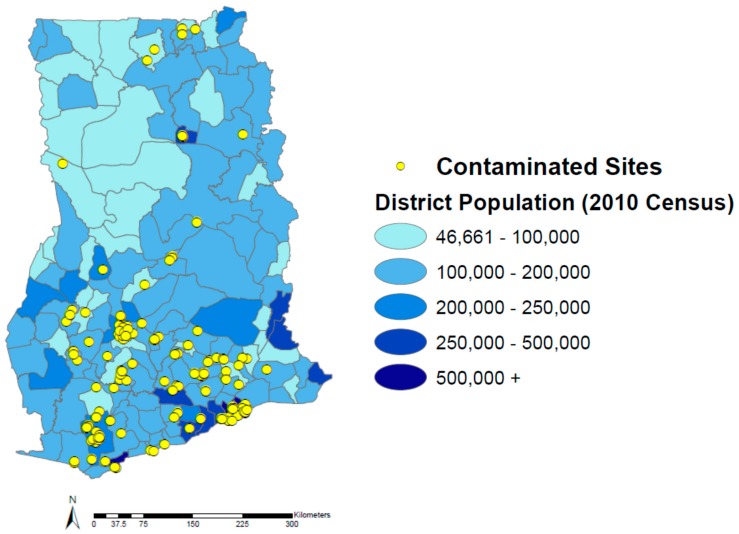
Population of Ghana by administrative district and contaminated sites.

There is at best a small positive correlation between population and contaminated sites at the administrative district level in Ghana (Figure 2; *ρ* = 0.285). In general, it can be assumed that a larger population would be highly correlated with more contaminated sites. However, because the areas of the districts vary greatly, a small correlation is to be expected. For example, a small district that is largely urban might have many more sites than a much larger district with a similar population. This is why it was not only important to review population, but also population density, or the number of inhabitants per kilometer squared.

**Figure 3:**
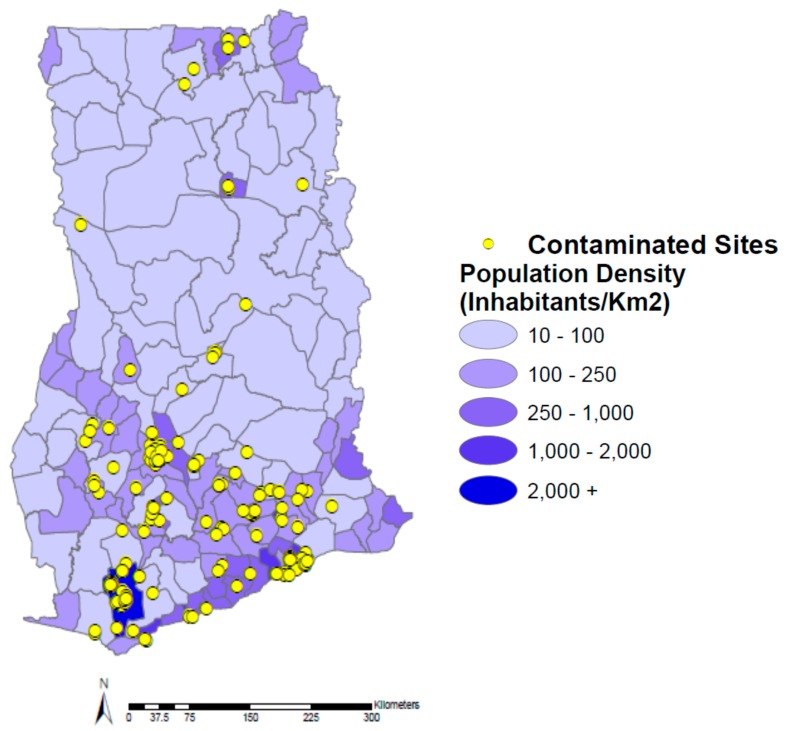
Population density of Ghana by administrative district and contaminated sites.

Correlations between 0.3 and 0.5 are generally thought of as low to medium strength correlations. Therefore, it appears that for the remaining four socio-demographic factors (population density, unemployment, education, and literacy rate), there is a low to medium correlation between them and contaminated sites at the administrative district level in Ghana. The medium positive correlation (*ρ* = 0.456) between population density and contaminated sites was unsurprising since it appears that the majority of sites can be found in largely urban districts in Figure 3 (e.g., Greater Accra and Kumasi Metropolitan). While it is apparent that there are significantly fewer sites in those districts with the lowest population densities (10–100 inhabitants/km^2^), the association is likely not stronger due to numerous districts with average population densities for Ghana (between 100 and 1000 inhabitants/km^2^). This could be explained by the fact that even though heavy industry is largely found in densely populated urban areas, several pollutants such as pesticides can be found much more frequently in rural or suburban areas.

**Figure 4:**
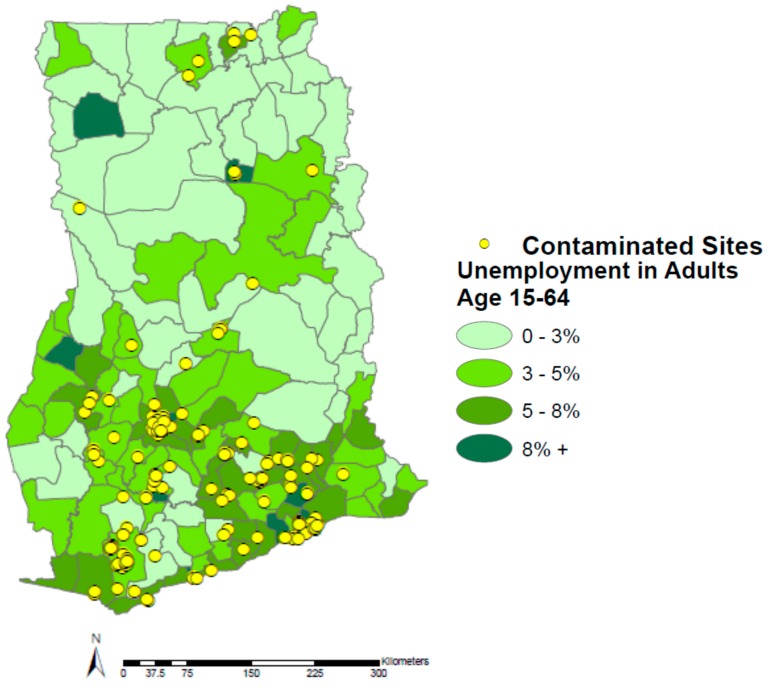
Unemployment in Ghana by administrative district and contaminated sites.

The highest correlation found in the analysis is the correlation between unemployment and contaminated sites at the administrative district level in Ghana (Figure 4; *ρ* = 0.478). Those districts with higher unemployment rates tend to have more contaminated sites than districts with lower unemployment rates. This is likely explained by the fact that “unemployment” is a relatively ambiguous term in Ghana and many other low- and middle-income countries. While the term typically denotes a lack of work in high-income countries, it is often synonymous with the informal sector in Ghana. Many unskilled laborers, for example, travel from the northern regions of the country to the capital in search of employment in industries such as scrap recycling and gold mining. These industries rely heavily on the informal sector, which could be skewing unemployment rates to urban areas with a greater number of contaminated sites. Unfortunately, this information is not currently available from GSS. However, unemployment has been used as a proxy for poverty in epidemiological studies, possibly explaining the relatively strong association [21]. While this is not a perfect proxy, because it does not take into account “the working poor”, it allows for a good estimate in a country like Ghana where unemployment typically denotes informal work. Furthermore, because work in many informal sector industries is characterized by low wages, little to no environmental or occupational health or safety standards, and temporary or transient conditions, many connections can be made between this factor and poverty [22].

**Figure 5:**
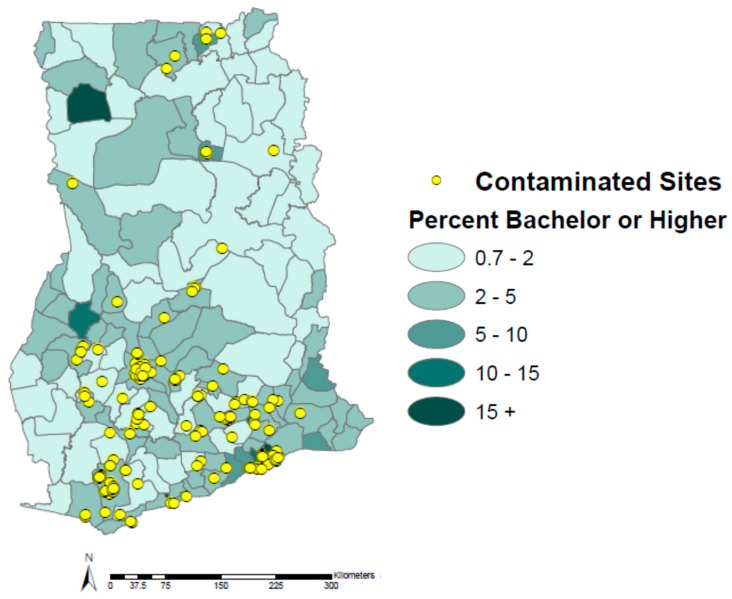
Education in Ghana by administrative district and contaminated sites.

**Figure 6:**
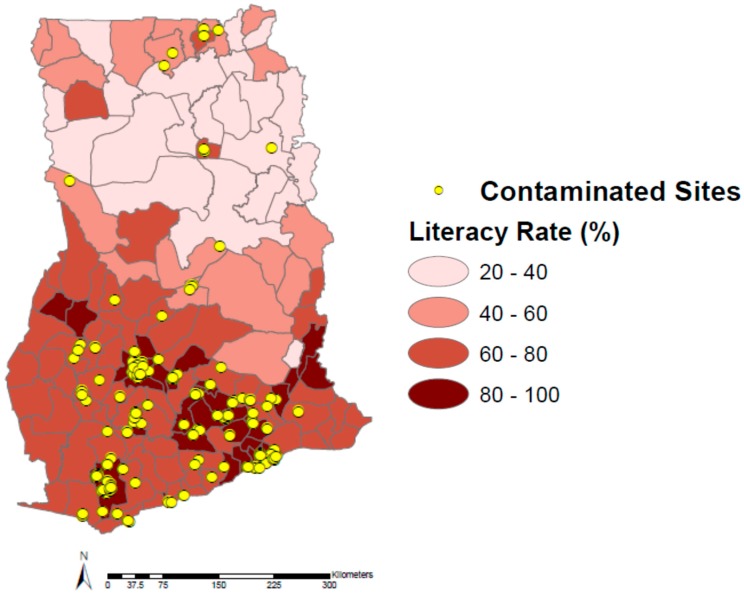
Literacy in Ghana by administrative district and contaminated sites.

Both education (bachelor degree or higher) and literacy rates have low to medium positive correlations to contaminated sites at the administrative district level in Ghana (*ρ* = 0.412 and 0.463, respectively). It is apparent from Figure 5 that there are at least two highly educated district outliers with no contaminated sites. However, in general, it appears that those districts with a greater percent of educated residents have more contaminated sites. This also appears to hold true for literacy rates in Ghana; those districts with higher literacy rates tend to have more contaminated sites (Figure 6). While the exact reasons for these associations are not known, it is likely due to the fact that Ghanaians in search of higher education frequently move to densely populated urban centers, such as Kumasi and Accra, for greater access to education and opportunities.

There are several important limitations of this paper that must be mentioned. First, while Pure Earth’s TSIP database likely remains the most comprehensive database of hazardous waste and contaminated sites in the world, the database is far from complete; there are certainly more sites in Ghana that have yet to be documented in the TSIP database. This is undoubtedly skewing the correlation between contaminated sites and our selected socio demographic factors. Therefore, this analysis is more on the associations between several socio demographic factors and available data on contaminated sites than a definitive assessment of correlations between the variables. Additionally, though it is unlikely, the methodology used to identify sites could be skewing the associations by assessing low-income areas more frequently than high-income areas. Pure Earth works with a highly experienced group of in-country experts that know the area well and are able to ascertain where contamination is most likely to occur rather than simply looking for contaminated sites in low-income or densely populated areas. This not only includes established factories and heavy industry sites but also informal and small-scale operations.

A second important limitation of this paper is the lack of socio demographic data at a micro level. It is possible that if the data could be broken down into subdivisions of administrative districts, we would find strong negative correlations between several of our selected factors and contaminated sites. For example: it is likely that within districts, residents that live directly adjacent to contaminated sites and industrial areas are less educated, have lower literacy rates, and higher rates of unemployment (or are employed in the informal sector). Unfortunately, GSS does not collect aggregated statistics at any administrative levels lower than the district level, and therefore subsequent analysis will be difficult.

## 4. Conclusions 

Several important conclusions can be made from this analysis. There appears to be a low to medium correlation between contaminated sites and the following socio demographic factors at the administrative district level in Ghana: higher population density, higher unemployment, greater education, and higher literacy rate. While the exact reasons for these correlations are not known, it is likely that population and industrial clustering in urban areas absent of adequate zoning regulations is partially responsible. Furthermore, the association between unemployment, which we believe acts as a proxy for poverty in this instance, and contaminated sites is particularly telling. These results support previous studies and suggest that certain socio demographic factors can be reasonably accurate predictors of contaminated site locations and perhaps used to develop predictive models of other contaminated sites.

Such identification of contaminated sites allows health and environmental ministries to better allocate scarce resources for improved health surveillance and remediation. Regular biomarker and environmental sampling in Ghana will not only ensure more complete data is collected but also help protect the health of Ghanaians. More data collection and research is needed at the village or neighborhood level to fully understand the associations between our selected socio demographic factors and contaminated sites.
