# Correction: Dowling, R., *et al*. Spatial Associations between Contaminated Land and Socio Demographics in Ghana. *Int. J. Environ. Res. Public Health* 2015, *12*, 13587–13601

**DOI:** 10.3390/ijerph13030252

**Published:** 2016-02-24

**Authors:** Russell Dowling, Bret Ericson, Jack Caravanos, Patrick Grigsby, Yaw Amoyaw-Osei

**Affiliations:** 1 Pure Earth, Formerly Blacksmith Institute, 475 Riverside Drive, Suite 860, New York, NY 10115, USA; Bret@pureearth.org (B.E.); Patrickgrigsby@gmail.com (P.G.); 2 School of Public Health, City University of New York, 2180 Third Ave., New York, NY 10035, USA; jcaravan@hunter.cuny.edu; 3 Green Advocacy Ghana, P.O. Box SK 482, Sakumono Estates, Tema, Ghana; wayoma59@hotmail.com

The author wishes to make the following correction to this paper [1]. Due to mislabeling, we replaced the original caption of Figure 1:

Figure 1. Regional map of Ghana. Source: MAGELLAN Geographics, 1992 [15].

with:

Figure 1. Regional map of Ghana. Source: Ezilon, 2009 [15].

The updated reference [2] for this figure is as follows:

15. Ghana Map—Political Map of Ghana. Available online: http://www.ezilon.com/maps/africa/ghana-maps.html (accessed on 12 February 2016).

The authors would like to apologize for any inconvenience caused to the readers by these changes.
